# Redefining Computational Enzymology with Multiscale Machine Learning/Molecular Mechanics: Catalytic Mechanism and Stereoselectivity in Diels–Alderases

**DOI:** 10.21203/rs.3.rs-8099572/v1

**Published:** 2025-11-25

**Authors:** Xujian Wang, Haocheng Tang, Xiongwu Wu, Bernard R. Brooks, Junmei Wang, Wan-Lu Li

**Affiliations:** † Aiiso Yufeng Li Family Department of Chemical and Nano Engineering, University of California San Diego, CA 92093, United States; ‡ Department of Pharmaceutical Sciences and Computational Chemical Genomics Screening Center, School of Pharmacy, University of Pittsburgh, Pittsburgh, Pennsylvania 15261, United States; ¶ Department of Computational and Systems Biology, School of Medicine, University of Pittsburgh, Pittsburgh, Pennsylvania 15261, United States; § Laboratory of Computation Biology, National Heart, Lung and Blood Institute, National Institutes of Health, Bethesda, MD, USA; ∥ Program of Materials Science and Engineering, University of California San Diego, CA 92093, United States

## Abstract

Enzymes catalyze complex chemical transformations with remarkable efficiency and selectivity, yet their atomistic mechanisms remain challenging to capture because conventional simulations trade accuracy for efficiency. Here we introduce a reactive machine learning/molecular mechanics (ML/MM) framework that bridges quantum chemistry with long-timescale sampling, enabling direct exploration of enzymatic transition states and free-energy landscapes. Coupled with metadynamics, this approach achieves nanosecond sampling of bond-forming reactions and quantitatively predicts activation barriers, mutational effects, and stereoselectivity. Applied to Diels–Alderases, the framework not only reproduces experimental activity and *endo/exo* preferences with sub-kcal mol^−1^ accuracy but also uncovers how pathway dynamics and local electrostatics preorganize substrates for selective outcomes. By uniting reactivity, conformational dynamics, and predictive power, this work establishes reactive ML/MM as a broadly applicable strategy for mechanistic enzymology and a foundation for the rational design of new biocatalysts.

## 1 Introduction

**Figure 1:**
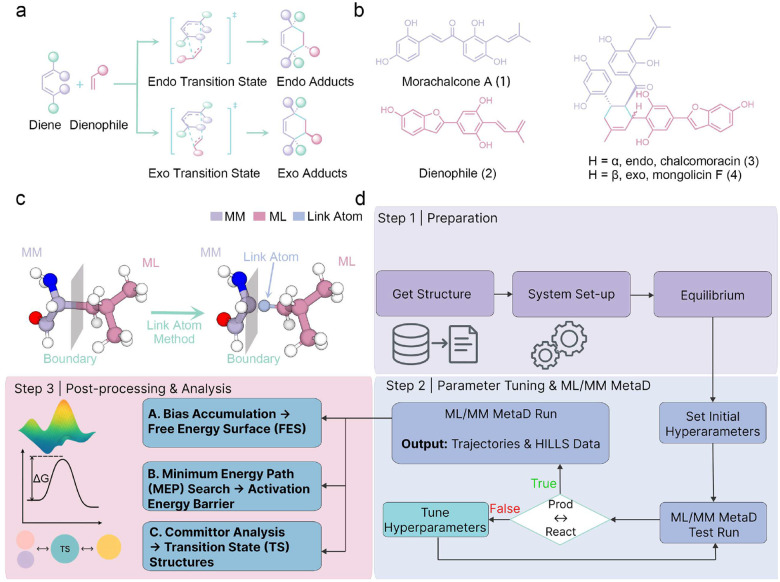
Diels–Alder reaction and reactive ML/MM MetaD workflow. **a**, Two competing pathways of the Diels–Alder reaction affording the *exo* and *endo* products. **b**, The natural substrate and catalytic products of the MaDA enzyme family. **c**, Schematic representation of the link-atom strategy within the ML/MM framework. **d**, The reactive ML/MM MetaD protocol, encompassing system setup, hyperparameter optimization for MetaD, and subsequent post-simulation analyses.

The Diels–Alder (D-A) reaction, a [4+2] cycloaddition between a conjugated diene and a dienophile, is a cornerstone of synthetic chemistry because of its efficiency in constructing complex cyclic structures. Recent studies have shown that nature also exploits this mode of reactivity, as numerous enzymes catalyzing D–A–like transformations have been discovered in biosynthetic pathways.^1–5^ Among them, SpnF was the first stand-alone intramolecular [4+2] cyclase identified,^6^ broadening the enzymatic repertoire of natural D–A reactions. More recently, Gao et al. reported the first intermolecular Diels–Alderase (MaDA) from *Morus alba*, which generates a methylcyclohexene skeleton, a motif prevalent in bioactive compounds of pharmaceutical interest.^1,2^ Despite its utility, MaDA exhibits poor enantioselectivity, limiting its broader biocatalytic potential (Fig. 1a). Protein engineering has since yielded two stereoselective variants: MaDA-1, which favors the *endo* pathway to produce chalcomoracin (**3**) from morachalcone A (**1**) and a dienophile (**2**), and MaDA-3, which instead directs the reaction along the *exo* pathway to yield mongolicin (**4**) (Fig. 1b). These complementary variants provide a compelling model system for dissecting the molecular basis of enzymatic stereocontrol and highlighting the challenges of controlling selectivity in complex enzymatic environments.

To move beyond trial-and-error experimental mutagenesis, computational methods provide atomic-level insights for rational enzyme design.^7,8^ Molecular dynamics (MD) and quantum mechanics (QM) are central tools: conventional molecular dynamics (cMD) relies on fixed force fields that cannot describe bond breaking, while QM captures such events but is too costly for large systems. To balance accuracy and efficiency, Walsh et al. introduced the hybrid QM/MM framework,^9^ which has been widely applied to the studies of mechanisms and free-energy landscapes, though its scope is limited by computational cost. Recent advances in machine learning interatomic potentials (MLIPs) reproduce QM-level accuracy at greatly reduced cost.^10–14^ Building on this, the ML/MM framework replaces the QM region with MLIPs, achieving both efficiency and accuracy, and has shown success in ensemble sampling,^13^ spectroscopy,^14^ and free-energy calculations.^15^ To extend ML/MM to chemical reactions, reactive MLIPs such as ANI-1xnr^16^ and AIMNet2-rxn^17^ have been developed. However, a remaining challenge is the ML/MM boundary, where severed covalent bonds introduce artifacts in electronic structure. While QM/MM offers remedies such as hybrid orbitals,^18^ buffer methods,^19^ and pseudobonds,^20^ their application in ML/MM is still limited. Among available boundary strategies, the link-atom method (Fig. 1c) remains the simplest and most practical choice,^21^ yet it has never been systematically implemented in ML/MM simulations of enzymatic reactivity. This absence has been a critical barrier, preventing ML/MM from extending beyond equilibrium dynamics to bond-making and bond-breaking processes.

Here we report the first incorporation of the link-atom method into a reactive ML/MM framework, implemented within AMBER.^22,23^ This advance establishes a robust strategy for treating covalent boundaries while enabling seamless coupling of reactive MLIPs with molecular mechanics environments. Specifically, the newly developed ANI-1xnr potential was implemented to model chemical reactions and metadynamics^24,25^ (MetaD) was integrated into the link-atom-compatible ML/MM framework (Fig. 1d), to successfully drive chemical transformations with extensive MD simulations at the nanosecond (ns) timescale. We validated the protocol on a recently discovered intramolecular Diels–Alder enzyme, where ML/MM MetaD reproduced the reaction barrier in agreement with both DFT calculations and experimental data. The actual transition state (TS) was obtained and confirmed through committor and frequency analyses, and the simulations further revealed that the MaDA-catalyzed D–A reaction is asynchronous, consistent with prior computational and experimental evidence.^1,2^ To demonstrate generality, we applied the framework to explore mutational effects on enantioselectivity and extended it to diverse substrates, successfully capturing differences in reaction barriers and stereochemical outcomes that were in agreement with the experimental observations.^1^ To support emerging reactive MLIPs, we further developed interfaces to EANN series,^26–28^ AIMNet series,^12,13,17,29^ SpookyNet^30^ and Egret-1^31^ in AMBER. Altogether, this implementation establishes a versatile ML/MM platform capable of resolving subtle effects of enantiomers, mutations, and substrates, thereby providing a robust tool for rational enzyme design.

## 2 Theory

### 2.1 Link-atom method for the ML/MM approach

Accurate multiscale simulations require proper treatment of covalent bonds crossing the ML/MM boundary. A straightforward partition can leave a dangling valence in the ML region, perturbing the electronic structure and introducing significant errors.^21^ To address this, we adapt the link-atom construction from QM/MM to ML/MM. For a bond split between atoms MLL (ML side) and MML (MM side), a link atom L is added to cap the ML valence. L introduces no additional degrees of freedom; its position is constrained along the MLL–MML bond vector and updated on the fly from their coordinates. Let $R_{\text{MLL}}$ and $R_{\text{MML}}$ be their Cartesian coordinates, and define $r=R_{\text{MML}}-R_{\text{MLL}}$, u=r/‖r‖. The link-atom position is then

(1) $$R_{L}=R_{\text{MLL}}+du=R_{\text{MLL}}+d\frac{R_{\text{MML}}-R_{\text{MLL}}}{\left\|R_{\text{MML}}-R_{\text{MLL}}\right\|},$$

where d is an equilibrium capping distance. Unless otherwise specified, we use a hydrogen link atom with d=1.09Å (the equilibrium C–H bond length in methyl). Because L is an auxiliary “ghost” atom used only to make the ML potential well-defined, it is not integrated as an independent particle; instead, $R_{L}$ is deterministically reconstructed at each step from Eq. (1).

The ML energy $E\left(R_{\text{ML}},R_{L}\right)$ depends on the ML-region coordinates and on $R_{L}$. The force on the link atom is

(2) $$F_{L}=-\frac{\partial E}{\partial R_{L}}.$$

To ensure energy-consistent coupling and momentum conservation, the contribution $F_{L}$ is redistributed to the boundary atoms by the chain rule, using the geometric dependence of $R_{L}$ on $R_{\text{MLL}}$ and $R_{\text{MML}}$:

(3) $$F_{\text{MLL}}^{\text{red}}=F_{L}\frac{\partial R_{L}}{\partial R_{\text{MLL}}},$$

(4) $$F_{\text{MML}}^{\text{red}}=F_{L}\frac{\partial R_{L}}{\partial R_{\text{MML}}}.$$

Here $F_{\text{MLL}}^{\text{red}}$ and $F_{\text{MML}}^{\text{red}}$ are the portions of the link-atom force that are accumulated on the two boundary atoms, and their sum reproduces the virtual work associated with $F_{L}$.

The required Jacobians follow directly from Eq. (1). Let I denote the 3 × 3 identity and set $r=R_{\text{MML}}-R_{\text{MLL}}$. Using $\partial u/\partial r=\|r{\|}^{-1}I-\|r{\|}^{-3}{rr}^{⊤}$, together with $\partial r/\partial R_{\text{MLL}}=-I$ and $\partial r/\partial R_{\text{MML}}=I$, we obtain:

(5) $$\frac{\partial R_{L}}{\partial R_{\text{MLL}}}=I-d\frac{I}{\left\|r\right\|}+d\frac{{rr}^{⊤}}{\|r{\|}^{3}},$$

(6) $$\frac{\partial R_{L}}{\partial R_{\text{MML}}}=d\frac{I}{\left\|r\right\|}-d\frac{{rr}^{⊤}}{\|r{\|}^{3}}.$$

These expressions are inserted into Eqs. (3)–(4) to compute the redistributed forces at each step. In practice, this construction (i) avoids introducing extra dynamical variables, (ii) preserves the correct projection of forces along and perpendicular to the cut bond, and (iii) stabilizes the ML description of the boundary without contaminating the MM region.

### 2.2 Energy partitioning at the ML/MM boundary

With the link-atom construction defined, we next specify the total energy decomposition to avoid double counting between the ML potential and the MM force field. We denote the atoms treated by the ML potential (capped by the link atom L attached to MLL) as ML and the remaining atoms including the boundary atom MML as MM. Unless stated otherwise, L is used only as an auxiliary coordinate within the ML energy and coupling terms.

#### Bonded terms.

All bonded interactions (bonds, angles, dihedrals) entirely within the ML region are excluded from MM calculations, since they are already represented by the ML potential. Only mixed terms that cross the ML/MM boundary and purely MM terms are retained:

(7) $$E_{\text{ML}-\text{MM}}^{\text{bonded}}=\underset{\begin{array}{c} \left(i,j\right)\in \text{bond}\\ \left\{i,j\right\}⊄\text{ML} \end{array}}{\sum }V_{\text{bond}}\left(i,j\right)+\underset{\begin{array}{c} \left(i,j,k\right)\in \text{angle}\\ \left\{i,j,k\right\}⊄\text{ML} \end{array}}{\sum }V_{\text{angle}}\left(i,j,k\right)+\underset{\begin{array}{c} \left(i,j,k,l\right)\in \text{dihedral}\\ \left\{i,j,k,l\right\}⊄\text{ML} \end{array}}{\sum }V_{\text{dihedral}}\left(i,j,k,l\right).$$

In particular, interactions involving MML and its MM neighbors are included, whereas those fully within the ML region (including the capped valence at MLL) are excluded from the MM Hamiltonian.

#### van der Waals (vdW) terms.

Nonbonded van der Waals (vdW) interactions between ML and MM atoms are evaluated at the MM level. While the interactions between any two ML region atoms including the auxiliary L, are omitted to avoid double counting with the ML energy. Denoting $R_{i}^{\text{MM}}$ and $R_{j}^{\text{ML}}$ as MM and ML coordinates, respectively, the interaction takes the standard Lennard–Jones 12–6 potential form:

(8) $$E_{\text{ML}-\text{MM}}^{\text{vdW}}=\underset{\begin{array}{c} i\in \text{MM}\\ j\in \text{ML} \end{array}}{\sum }\epsilon_{ij}\left[{\left(\frac{A_{ij}}{\left\|{\text{R}}_{i}^{\text{MM}}-{\text{R}}_{j}^{\text{ML}}\right\|}\right)}^{12}-{\left(\frac{B_{ij}}{\left\|{\text{R}}_{i}^{\text{MM}}-{\text{R}}_{j}^{\text{ML}}\right\|}\right)}^{6}\right],$$

where $A_{ij}$, $B_{ij}$ and $\epsilon_{ij}$ are given by standard mixing rules and depend on the atom types of i and j.

#### Electrostatics.

For Coulomb coupling we adopt an energy-consistent protocol following Field and co-workers,^32^ combined with a charge-balancing scheme similar to Walker and colleagues for stable QM/MM embedding.^21^ The ML region is assigned an integer total charge; if its nominal charge is non-integer, a user-specified integer is enforced by redistributing small compensating charges onto MM atoms to maintain overall neutrality (or the desired total charge). When calculating the coupling energy, purely ML–ML electrostatics (including pairs involving L) are excluded, and ML–MM interactions are evaluated at the MM level. For the MM boundary atom MML, its coordinate is replaced by the link-atom position $R_{L}$ when computing distances to other MM atoms, thereby reducing artifacts from cutting a polar bond at the boundary. The resulting coupling term is:

(9) $$E_{\text{ML}-\text{MM}}^{\text{elec}}=\underset{\begin{array}{c} i\in \text{MM},i\neq \text{MML}\\ j\in \text{ML} \end{array}}{\sum }\frac{q_{i}q_{j}}{\left\|{\text{R}}_{i}^{\text{MM}}-{\text{R}}_{j}^{\text{ML}}\right\|}+\underset{j\in \text{MM},j\neq \text{MML}}{\sum }\frac{q_{\text{MML}}q_{j}}{\left\|{\text{R}}_{L}-{\text{R}}_{j}^{\text{MM}}\right\|},$$

where $q_{i}$ and $q_{j}$ are the MM partial charges. This formulation avoids double counting with the ML energy, ensures a consistent electrostatic boundary, and improves numerical stability in practice.

## 3 Results and discussion

### 3.1 Probe the MaDA Diels–Alderases with ML/MM MetaD

**Figure 2:**
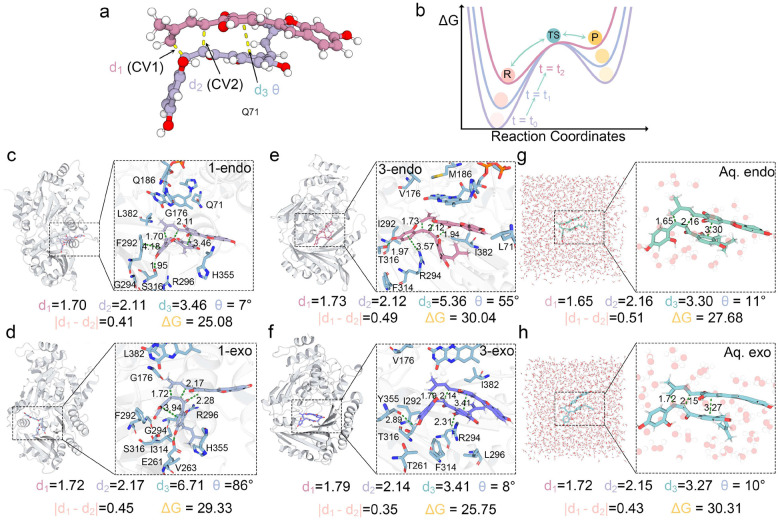
Transition states from ML/MM MetaD. **a**, CVs and key structural metrics: $d_{1}$ and $d_{2}$ correspond to the two forming C–C bonds in the Diels–Alder reaction, which are also employed as CVs in MetaD; $d_{3}$ denotes the centroid–centroid distance between the two benzene rings; and θ represents the inter-ring dihedral angle. **b**, Schematic depiction of how MetaD accelerates the reaction by filling the activation-energy barrier with bias potentials. TSs were identified from structures exhibiting bidirectional transitions between reactants and products. **c-h**, Representative TS conformations determined for MaDA-1, MaDA-3, respectively, and the aqueous environment, each resolved for both *exo* and *endo* configurations, with the corresponding structural metrics indicated below. All distances are reported in Å, and all energies are given in k cal mol^−1^.

To model the MaDA-catalyzed D–A reaction, we employed a reactive ML/MM MetaD combining ANI-1xnr with explicit environmental sampling. ANI-1xnr was chosen because, unlike many general-purpose MLIPs, it is specifically curated to describe reaction pathways. MetaD was then integrated to overcome the challenge of rare-event sampling, accelerating transitions along well-defined collective variables (CVs). For the intramolecular D–A cycloaddition, as shown in Fig. 2a, we employed two CVs corresponding to the forming C–C bonds, a choice that accounts for the asynchronicity of this reaction.^2^ Their suitability was validated by path-CV steered MD simulations, which revealed a sequential mechanism in which CV1 is elaborated first, followed by CV2, and finally the products dissociate into two substrates (Fig. S2).

Using these CVs and optimized MetaD parameters, we investigated MaDA-3, MaDA-1, and the aqueous reaction of substrates **1** and **2**. The predicted activation free energies $\left(\Delta G^{‡}\right)$ reproduced the relative trends observed in DFT and experiment, though absolute values exhibited a positive offset about 5 kcal mol^−1^ compared to theozyme and experimental results.^1,2^ Such deviations may also reflect (i) the generalization limits of MLIPs trained on finite DFT datasets, (ii) incomplete treatment of long-range interactions, and (iii) the use of mechanical rather than electrostatic embedding. However, the traditional methods, like theozyme model would also leads to energy difference up to 5 kcal mol^−1^,^33–37^ but for a different reason: their oversimplified treatment of the protein environment inherently neglects conformational ensembles, electrostatics, and mutational effects. By contrast, our ML/MM MetaD framework systematically incorporates protein dynamics and explicit environment, enabling reliable prediction of relative barriers. More importantly, it correctly capture enantioselectivity trend inaccessible to static theozyme models: MaDA-3 favors the *exo* product (**4**), MaDA-1 favors the *endo* product (**3**), and aqueous solution favors the *endo* pathway. The computed relative activation free energy (ΔΔG) values are in excellent agreement with the DFT results (Table S2), supporting its robustness as a more meaningful metric than absolute $\Delta G^{‡}$.

Importantly, despite the systematic offset in $\Delta G^{‡}$, relative barriers are reliably captured by error cancellation. Our ML/MM MetaD framework correctly distinguishes enantioselective outcomes: MaDA-1 favors the *endo* product (**3**), whereas MaDA-3 favors the *exo* product (**4**). In aqueous solution, where the environment is more disordered and steric constraints are reduced, the *endo* pathway is thermodynamically preferred. In this case, our method yields $\Delta \Delta G=2.63\;\text{kcal}\;{\text{mol}}^{-1}$, in close agreement with the DFT estimate of 2.8 kcal mol^−1^ (Table S2). Likewise, the difference between MaDA-3 *exo* and MaDA-1 *endo* is reproduced with high fidelity (ML/MM: 0.67 kcal mol^−1^; DFT: 0.5 kcal mol^−1^, Table S2). Taken together, these results indicate that ΔΔG is a more reliable and mechanistically meaningful metric than absolute $\Delta G^{‡}$ within this protocol.

Beyond activation free energies, ML/MM MetaD enables direct and reliable identification of TS structures, a key requirement for structure-based enzyme design. Its computational efficiency supports nanosecond-scale simulations, during which multiple reactive events were sampled (Fig. 2b), providing access to well-sampled ensembles of TS configurations, in contrast to the isolated snapshots typically obtained from static QM or theozyme models. Committor and vibrational frequency analyses (Fig. S5) confirmed the validity of the generated configurations. To characterize TS geometry, we defined and evaluated four geometric descriptors shown in Fig. 2a: the two forming C–C distances $\left(d_{1},d_{2}\right)$, the benzene–benzene centroid distance $\left(d_{3}\right)$, and the dihedral angle between the ring planes (θ). Interestingly, $d_{1}$ and $d_{2}$ varied only slightly (1.65–1.79 Å and 2.11–2.17 Å), making them less informative. In contrast, $d_{3}$ and θ are better geometric descriptors for distinguishing favored enantiomers in MaDA-1 and MaDA-3 (Figs. 2C–F), as they closely resembled aqueous-phase TS structures (Figs. 2G and H). Favored TSs consistently displayed stacked benzene rings, stabilized by π-π interactions. By contrast, in disfavored TS structures (Figs. 2D and E), this stacking arrangement was disrupted, highlighting a key structural determinant underlying the distinct enantioselective outcomes of MaDA-1 and MaDA-3. Interestingly, disfavored TSs often attempted to compensate through alternative interactions. For example, in the MaDA-1 *exo* pathway, one benzene ring distorted the backbone of substrate **1** to establish a weak hydrogen bond with E261 and another π-π stacking interaction (Fig. 2d). However, this could not compensate for the loss of stabilization normally provided by F292 and S316 (Fig. 2c). A similar pattern was observed in MaDA-3, where alternative contacts arose but remained insufficient. Importantly, the model was able to capture higher-order weak interactions, such as multipole effects, π-π stacking, and π-cation interactions, which are typically not well described by traditional force fields due to their limited functional form.^38,39^ This finding highlights the potential of ML/MM approaches in accurately describing delicate electrostatic interactions. It also demonstrate that ML/MM MetaD framework not only identifies preferred TS geometries but also elucidates the compensatory interactions that arise in disfavored enantiomeric pathways, which prove insufficient to overcome their higher free-energy cost.

Apart from stereoselectivity, the MaDA-catalyzed D-A reaction exhibits a pronounced asynchronicity in bond formation, with the difference between the two forming C-C bonds $\left(d_{1}-d_{2}\right)$ spanning 0.35 to 0.51 Å. This agrees with previous DFT studies and kinetic isotope effect (KIE) experiments on substrate analogs,^2^ which together established asynchronicity as a key mechanistic signature of D–A enzymology. Motivated by these findings, we tested whether ML/MM MetaD could emulate KIEs computationally. By substituting hydrogen (1.008 amu) with deuterium (2.014 amu), our simulations reproduced experimental KIE trends (Fig. S3). These results demonstrate that ML/MM MetaD not only recapitulates stereoselectivity and barrier heights but also extends to subtle kinetic observables such as isotope effects, underscoring its potential as a broadly predictive tool for enzymatic reaction mechanisms.

### 3.2 Investigation of Mutation Effects on Activity and Stereoselectivity

**Figure 3:**
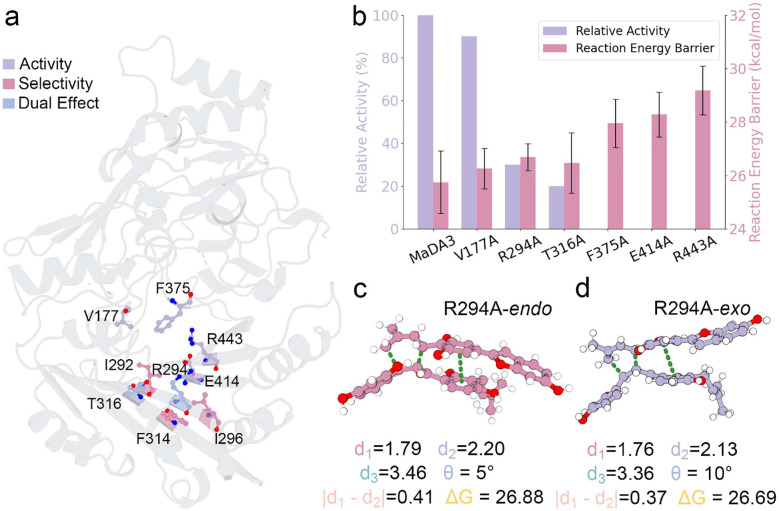
Mutation effects on activity and selectivity. **a**, Key residues identified as critical determinants of catalytic activity and stereoselectivity. **b**, Experimentally determined relative activities correlated with computationally estimated activation-energy barriers ($\Delta G^{‡}$, in kcal mol^−1^); error bars represent the standard error. **c** and **d**, Structural paradigm illustrating the impact of the R294A mutation on both catalytic activity and product selectivity, with key distances reported in Å.

The information obtained from ML/MM MetaD simulations on the wild type motivated us to examine whether this protocol could be applied to mutants, thereby probing how amino acid substitutions affect catalytic activity and stereoselectivity. Several residues located near the catalytic site were selected (Fig. 3a), as these first-shell residues are expected to strongly influence the TS structures.

For catalytic activity, we compared the predicted active free energies with experimentally measured relative activities of MaDA mutants (Fig. 3b). A clear relationship was observed that variants with higher $\Delta G^{‡}$ exhibited reduced activity, consistent with the principle that increased energetic barriers diminish catalytic turnover. In the experimental assay, reactions were quenched after a short incubation period,^1^ such that variants with $\Delta G^{‡}$ exceeding a critical threshold yielded no detectable product. This trend was reproduced in our simulations, which predicted complete loss of activity for F375A, E414A, and R443A, all of which exhibited substantially higher barriers than the wild type or other mutants. Moreover, when considering the full panel of variants, the computed $\Delta G^{‡}$ values showed a strong linear correlation with experimentally measured relative activities, underscoring the ability of ML/MM MetaD to connect mutational effects at the atomic scale with macroscopic measures of enzymatic efficiency.

We further investigated the stereoselectivity by calculating the ΔΔG values. Consistent with previous experimental studies that identified stereodetermining residues,^1^ MaDA-1 is intrinsically endo-selective, whereas MaDA-3 favors the exo product. To probe the molecular origins of this preference, we simulated a series of MaDA-3 mutants: R294G, R294A, MU3 (I292F, R294G, L296R), and MU5 (MU3 plus F314I and T316S), that mirror variants previously characterized experimentally. These substitutions progressively reduced the *exo* preference, with MU5 displaying an ~1:2 *exo:endo* ratio and largely losing stereoselectivity. Although direct $\Delta G^{‡}$ measurements were not available, ΔΔG values could be inferred from product ratios via the Eyring equation.^40^ Our ML/MM MetaD simulations reproduced these values with sub-kcal mol^−1^ accuracy on average, as listed in Table S3. This level of agreement highlights the promise of ML/MM MetaD for resolving subtle free-energy differences between enantiomeric pathways and predicting how mutations reshape enzyme stereoselectivity.

Importantly, residue R294 is identified as a critical determinant that modulates both catalytic activity and stereoselectivity. Mutation of R294 to alanine reduced the activity to nearly 30% of the wild type (Fig. 3c) and shifted the product distribution from exclusively *exo* to an *endo:exo* ratio of 1:1.38 (Table S3). Consistent with the TS configurations observed in Fig. 2f, this result highlights the essential role of the π–cation interaction contributed by R294 in stabilizing the transition state. Notably, neighboring residues appear capable of reorganizing their side chains to conpensate sterically, leading to minimal differences in the geometric descriptors of $d_{3}$ and θ between the two TS structures (Fig. 3c and d), which remain key parameters for enantiomer discrimination.

### 3.3 Exploring the Suitability of MaDA Enzymes Across a Broad Substrate Spectrum

**Figure 4:**
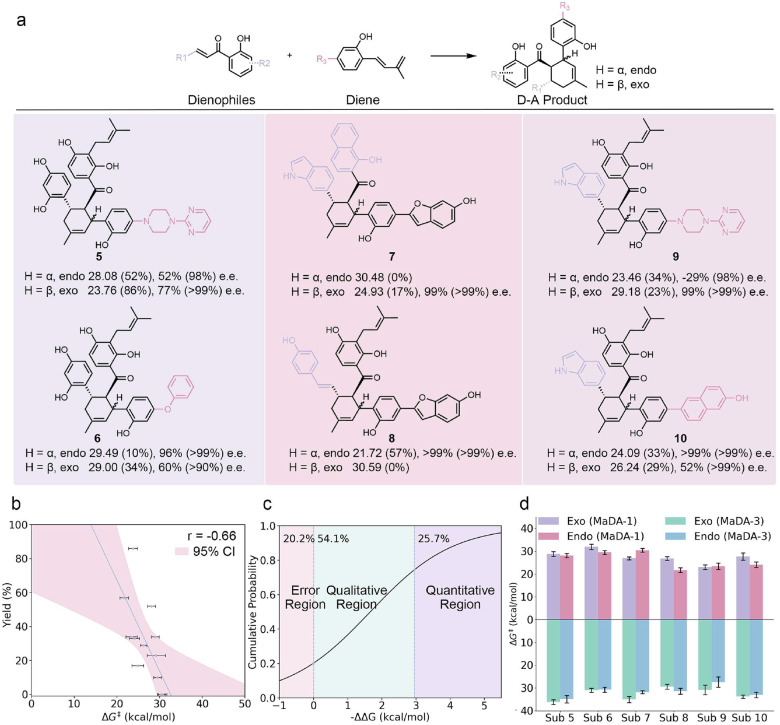
Generalizability of ML/MM across a broad catalytic substrate spectrum. **a**, Estimated activation-energy barriers (in kcal mol^−1^) and corresponding enantiomeric excesses (e.e.) obtained from ML/MM simulations; experimental yields and e.e. values are shown in brackets. **b**, Linear regression of experimental yields versus estimated $\Delta G^{‡}$, with data points shown as dots and error bars representing the standard error. The absolute Pearson correlation coefficient (r) and the 95% confidence interval (CI) are indicated. **c**, Analysis and empirical cumulative distribution function fitting of the -ΔΔG distribution. The error region denotes cases where the predominant enantiomer is incorrectly assigned (-ΔΔG<0); the qualitative region corresponds to correct enantiomer prediction with > 1% deviation in e.e. relative to experiment; and the quantitative region indicates both correct enantiomer assignment and quantitative agreement in e.e. values. **d**, Computed activation-energy barriers (in kcal mol^−1^) in MaDA-1 and MaDA-3 for the two enantiomeric pathways.

Enzymes typically catalyze not only their natural substrates but also structurally related analogs. Understanding the substrate spectrum is therefore essential for assessing catalytic potential and guiding future industrial applications. We therefore performed virtual screening using our ML/MM MetaD protocol to estimate $\Delta G^{‡}$ and enantiomeric selectivities based on ΔΔG across a panel of substrate variants. Because the intramolecular D-A reaction involves two reacting fragments, both diene and dienophile functional groups were systematically modified as depicted in Fig. 4a, along with more extensive dual substitutions exemplified by substrates **9** and **10**.

As expected, $\Delta G^{‡}$ values showed a clear correlation with experimental yields and ΔΔG values accurately reflected enantiomeric excess (e.e.), as illustrated in Fig. 4a. This agreement across a diverse substrate panel underscores the strength of the ML/MM MetaD framework in capturing both catalytic activity and stereoselectivity. The only deviation was substrate **7**-*exo*, which displayed a low $\Delta G^{‡}$ yet poor yield. This discrepancy likely arises from altered binding or release modes introduced by multiple functional group substitutions, effects beyond what is represented by barrier heights alone. Importantly, such cases highlight the complementary roles of kinetics and binding dynamics in catalysis, rather than limitations of the ML/MM MetaD framework itself. Overall, the correlation between $\Delta G^{‡}$ and yield remained strong (r=-0.66, Fig. 4c), underscoring the robustness and predictive power of our approach.

Stereoselectivity was also well reproduced by our protocol, even though ΔΔG predictions are intrinsically sensitive to small free-energy differences, with an e.e. of 98% corresponding to only 2.95 kcal mol^−1^ difference. Across the tested substrate panel, approximately 25% of cases achieved full quantitative agreement with experiment, while an additional 54% were qualitatively correct, capturing the dominant enantiomer. This level of accuracy is notable given the sub-kcal mol^−1^ precision required to resolve stereochemical outcomes. The only clear deviation occurred for substrate **9**-*endo*, where the preferred enantiomer was reversed; however, the computed energy difference was only 0.78 kcal mol^−1^ (Fig. 4d), a value well within the accepted uncertainty range of state-of-the-art free-energy simulations. Importantly, such small deviations, while magnified when expressed as enantiomeric excess, do not undermine the broader predictive capacity of the method. Instead, they highlight both the inherent difficulty of stereoselectivity prediction and the strength of the present approach, which achieves reliable performance across a chemically diverse substrate set.

## 4 Conclusion

Understanding enzymatic catalysis at the atomistic level is essential for enzyme engineering and industrial application, yet traditional computational approaches (MMFF, theozyme models, QM/MM) face intrinsic trade-offs between accuracy and efficiency. To overcome these limitations, we developed a unified multiscale framework that integrates reactive MLIPs into AMBER with a link-atom boundary scheme, enabling accurate and efficient description of chemical reactivity in enzymatic systems. Coupled with metadynamics, this ML/MM framework achieves nanosecond-timescale simulations that directly identify transition states in the MaDA family and in aqueous solution. It not only reproduces experimental and DFT trends in $\Delta G^{‡}$ and ΔΔG, but also reveals stereoselectivity mechanisms arising from aromatic stacking geometries and clarifies how key residues such as R294 exert dual control over activity and selectivity. Moreover, virtual screening across diverse substrates further demonstrated strong correlations between $\Delta G^{‡}$ and experimental yields, while ΔΔG predictions reached sub-kcal mol^−1^) accuracy in enantiomer discrimination. Together, these results underscore the generality of the framework for probing activity, selectivity, mutational effects, and substrate scope.

Although further advances in reactive MLIP development and embedding strategies will be needed to extend ML/MM to radical, metalloenzyme, and photoenzyme catalysis, the present framework already bridges physics-based accuracy with machine-learning efficiency. By unifying chemical reactivity, environment effects, and statistical sampling in a single platform, it offers a powerful route to rational enzyme design and and paves the way toward next-generation AI- and physics-informed computational enzymology.

## 6 Methods

### General settings.

In this study, MD simulations were conducted using our ML/MM module implemented in the molecular simulation engine SANDER, based on the AMBER23 source code.^22,23^ All calculations were performed on NVIDIA L40s GPUs and Intel Xeon Platinum 8462Y+ processors.

The protein was described with the AMBER14SB force field,^41^ and the TIP3P model^42^ was used for water molecules. Notably, the MaDA family contains a non-natural residue, a histidine covalently bound to flavin adenine dinucleotide (FAD). This covalently linked fragment was extracted, and the broken valence formerly connected to the neighboring residue was capped with acetyl and methylamine groups. The geometry of the capped structure was optimized using Gaussian^43^ at the $\omega \text{B}97\text{X}/6-31{\text{G}}^{*}$ level,^44,45^ and RESP charges^46^ were derived at the HF/6–31G* level and ensure an integer total charge. The remaining parameters were assigned with GAFF2.^47^ Ligands were parameterized with GAFF2, and partial atomic charges were obtained using the RESP method in *Antechamber*.

Systems were built with *tleap* (AMBERTOOLS23), solvated in a TIP3P water box with at least 10 Å padding between solute and box edges. Na^+^ and Cl^−^ ions were added to neutralize the system and adjust the salt concentration to 0.15 M.

All simulations began with energy minimization, consisting of 1,000 cycles of steepest descent followed by 9,000 cycles of conjugate gradient, for a total of 10,000 cycles. Bond lengths involving hydrogen atoms were constrained using the SHAKE algorithm,^48^ and a nonbonded cutoff of 9.0 Å was applied. Minimization was considered converged when the root-mean-square gradient of the potential energy fell below 0.0001 kcal mol^−1^ Å^−1^.

Equilibration was carried out under cMD conditions without MLIPs, with an integration timestep of 1 fs. Simulations were run for 100,000,000 steps (100 ns). The temperature was maintained at 323 K, consistent with experimental conditions,^1^ using a Langevin thermostat with a friction coefficient of 5.0 ps^−1^. Pressure was maintained at 1.01325 bar using the Berendsen barostat with a relaxation time of 2.0 ps. Periodic boundary conditions were applied, and a 9.0 Å cutoff was used for van der Waals and electrostatic interactions.

### Selection of the ML Region.

The ML region included residues 71, 176, 186, 261, 263, 292, 294, 296, 314, 316, 355, and 382, which have been experimentally demonstrated to play critical roles in catalytic activity and stereoselectivity.^1^ The substrates were also included in the ML region, while the remaining parts of the system were treated with the MMFF. Residue M186 in MaDA3 was excluded because the ANI-1xnr potential currently does not support sulfur atoms.

### CV validation with steered MD.

Prior to metadynamics, CVs were validated using the path CV^49^ formalism combined with steered molecular dynamics^50^ (SMD). Two forming C–C bond distances were employed as descriptors to define the reaction pathway. The path CV was expressed as a weighted combination of reference structures along the reaction coordinate:

$$s=\frac{\sum_{i=1}^{N}i\;\text{exp}\;\left(-\lambda R\left[X-X_{i}\right]\right)}{\sum_{i=1}^{N}\;\text{exp}\;\left(-\lambda R\left[X-X_{i}\right]\right)},\;z=-\frac{1}{\lambda }\text{ln}\;\left[\sum_{i=1}^{N}\;\text{exp}\;\left(-\lambda R\left[X-X_{i}\right]\right)\right],$$

where $R\left[X-X_{i}\right]$ is the mean-square deviation between the instantaneous configuration X and the $i^{\text{th}}$ reference structure $X_{i}$, s measures the progression along the path, and z quantifies the distance from the path.

SMD was performed along the s coordinate with a moving restraint, in which the target value of s was gradually increased from 0.0 to 100.0 over the course of the simulation. A short equilibration stage of 100,000 steps was applied prior to pulling (STEP0), and an additional relaxation stage was included after reaching the final target (STEP1). A harmonic tube potential with a force constant of 200 kcal mol^−1^ Å^−2^ was applied on the z coordinate to confine sampling near the predefined path. Each simulation was carried out for a total of 2,000,000 steps with a timestep of 0.5 fs, corresponding to 1 ns of sampling, under the same thermodynamic conditions as cMD.

### Metadynamics.

ML/MM MetaD simulations were initiated from equilibrated structures, under conditions nearly identical to those used in cMD, except that a smaller timestep of 0.5 fs was applied. Each simulation was run for 2,000,000 steps, corresponding to 1 ns of sampling. All simulations employed plumed-2.9^51,52^ compiled with AMBER.

Well-tempered metadynamics (WT-MetaD) was carried out by plumed using two CVs. For both CVs, an upper-wall restraint was imposed at 3.0 Å with a force constant of 50 kcal mol^−1^ Å^−2^ to prevent exploration of irrelevant regions of the free-energy landscape. Gaussian bias potentials were deposited every 50 MD steps (PACE = 50), with an initial height of 0.6 kcal mol^−1^ and a width of 0.08 Å for each CV. For each data point, ML/MM MetaD simulations were performed in five independent replicates, and the reported values represent the averages over these runs.

### Free-Energy Surface Reconstruction and Minimum-Energy Pathway Identification.

The free-energy surface (FES) was reconstructed using the sum_hills module of plumed, based on the HILLS files collected from MetaD simulations. Gaussian kernels were accumulated onto a 300 × 300 grid for $d_{1}$ and $d_{2}$ over the range of 1.4–3.5 Å, and the global minimum was set to zero as the reference energy.

From the resulting grid, the lowest free-energy pathway (LFEP) was identified using the LFEP algorithm.^53^ The highest point along this pathway was taken as the TS.

### Committor Analysis.

According to the minimum-energy pathway, cpptraj was used to identify candidate structures corresponding to the most probable transition states by locating configurations with the root-mean-square deviation (RMSD) less than 0.1 Å in the $d_{1}$ and $d_{2}$ distances, defined as

$$\text{RMSD}=\sqrt{\frac{1}{N}\sum_{i=1}^{N}{\left(d_{i}-d_{i}^{\text{ref}}\right)}^{2}}.$$

From each candidate structure, ML/MM MD simulations were restarted for 10,000 steps with a timestep of 0.5 fs. For each structure, ten independent simulations were performed, and the trajectories were analyzed collectively. A structure was considered a TS if the corresponding simulations demonstrated connectivity to both reactant and product states.

### Frequency Analysis.

Frequency calculations were performed on the TS structures by extracting the ML region, with link atoms placed according to Eq. 1. Energies, forces, and the Hessian matrix were evaluated using ANI-1xnr with PyTorch autograd.^54^ The gradient and Hessian were obtained from first- and second-order derivatives of the total energy,

$$g_{i}=\frac{\partial E}{\partial x_{i}},\;H_{ij}=\frac{\partial^{2}E}{\partial x_{i}\partial x_{j}}.$$

To remove the dependence on nuclear masses, the Hessian was transformed into its mass–weighted form,

$${\tilde{H}}_{ij}=\frac{H_{ij}}{\sqrt{m_{i}m_{j}}},$$

which was subsequently diagonalized to obtain eigenvalues $\lambda_{k}$ and eigenvectors. The vibrational frequencies were then calculated directly from the eigenvalues as

$$\nu_{k}=\frac{1}{2\pi c}\sqrt{\lambda_{k}},$$

where c is the speed of light.

## Supplementary Material

This is a list of supplementary files associated with this preprint. Click to download.

- MLMMSI.pdf

## Data Availability

The software developed in this work is available at https://github.com/ClickFF/MLMM4AMBER.
